# Climate-related stressors, community healthcare systems, and adaptation strategies: A scoping review

**DOI:** 10.1016/j.joclim.2025.100574

**Published:** 2025-11-05

**Authors:** Sonyta Saad, Chrisgone Adede, David Citrin, Beatrice Wasunna, Mourice Barasa, Krishna Jafa, Kristie L. Ebi

**Affiliations:** a Center for Health and the Global Environment (CHanGE), University of Washington, Seattle, WA, USA; b Medic Inc, Hekima University College, Riara Lane, off Riara Road, Nairobi, Kenya; c Medic Inc, Fillmore St, San Francisco, USA; d Department of Global Health, University of Washington, Seattle, WA, USA; e Department of Anthropology, University of Washington, Seattle, WA, USA

**Keywords:** Climate-related stressors, Climate change, Climate variability, Extreme weather community health, Lmics, Machine learning, Artificial intelligence

## Abstract

**Introduction:**

Climate-related stressors are a global challenge with effects extending far beyond the environment, significantly impacting the provision of healthcare services, especially in the African and Asian countries selected for examination in this scoping review. Climate-related stressors are expected to significantly increase health risks in these countries, continuing to disproportionately affect vulnerable groups.

**Methods:**

We conducted a scoping review to identify, select, and synthesize relevant literature on climate-related vulnerability factors, exposure pathways and interventions in the geographic regions where the nonprofit organization Medic (www.medic.org) operates. We also reviewed published literature that explored the use of Artificial Intelligence (AI) as a potential tool for community health systems to adapt to and mitigate climate-sensitive health outcomes.

We searched PubMed, Google Scholar, Scopus, and Web of Science.

**Results:**

The search generated 3184 studies available in English since 2000. Overall, 96 studies were screened for inclusion, resulting in 30 studies selected for the review, including 4 reports and articles from gray literature. Overall, the review identified critical gaps in the literature on community-level health interventions to reduce climate-sensitive health risks.

**Discussion:**

The current literature is skewed toward community perception of climate-sensitive health outcomes and adaptation challenges faced by the health system. Results similarly revealed gaps in published literature on adaptation interventions in community health, and healthcare delivery more broadly, that employ AI.

There remains a need for more evidence on the potential use of AI for future interventions to address the growing threat of climate variability to community healthcare and climate-sensitive health outcomes.

## 1 Introduction

Climate-related stressors (Climate change, climate variability, and extreme weather) are a global challenge, and their effects extend far beyond environmental concerns, significantly impacting the provision of healthcare services. This is particularly observed in a number of countries where the nonprofit Medic operates in Africa and Asia, including Burundi, Democratic Republic of Congo, Ghana, Kenya, Malawi, Mali, Niger, Republic of South Africa, Togo, Uganda, Zanzibar (Tanzania), Zimbabwe, Nepal, India, and the Philippines.

Referencing the World Bank Group’s classification of countries on the basis of income, these countries fall in a range from Lower-Middle income to Low-income countries, with one Upper-Middle income country, the Republic of South Africa [1]. Climate-related stressors are expected to significantly increase health risks in these countries, disproportionately affecting vulnerable groups [2]. Many of these countries do not have the infrastructure to adequately prepare for and respond to the diverse challenges posed by climate-related stressors. Some face limited access to advanced healthcare technologies, essential medical supplies, and well-equipped healthcare facilities, making it difficult to cope with growing health crises exacerbated by climate-related stressors.

The geographic regions where Medic has active deployments of the Community Health Toolkit (CHT) [3] are more vulnerable to the risks of climate variability due to their high dependence on natural resources and agriculture [4]. For example, Burundi and Malawi are more susceptible to extreme weather events, prolonged droughts, and unpredictable precipitation patterns [5].

The resultant disruption to livelihoods can lead to increased poverty and food insecurity, further straining the healthcare system. These nations frequently struggle with high rates of infectious diseases such as malaria and dengue fever, with climate-related stressors acting as a stress multiplier, enhancing the transmission and altering the geographic distribution and seasonality of certain vector borne diseases [6]. The Intergovernmental Panel on Climate Change (IPCC) states that the risks from vector borne diseases are projected to increase with warming temperatures [7].

Extreme weather events can also lead to the displacement of populations, limit access to healthcare services, and change the overall social and environmental determinants of health [2]. Limited access to safe water and sanitation facilities in many of these countries further exacerbates the health risks, as waterborne diseases become more prevalent. Studies have also shown pathways through which climate change and natural disasters impact the psychological and mental well-being of vulnerable populations, often manifesting in post-traumatic stress disorder, depression, and acculturation anxiety among climate refugees [8].

The geographical locations of these countries make them particularly susceptible to climate-related disasters, such as hurricanes, floods, and wildfires. Coastal nations can struggle with the direct impacts of rising sea levels, exposing vulnerable communities to increased risks of flooding and storm surges [9]. The lack of robust emergency response systems in some of these countries further complicates the ability to manage health crises effectively during and after such disasters. While the specific vulnerability factors and exposure pathways may differ, the need for urgent interventions to address these challenges and mitigate their impact on community health is a common imperative.

Medic is a non-profit organization that designs, delivers, and supports open-source software for health workers providing care in the world’s hardest-to-reach communities. In collaboration with the University of Washington Center for Health and the Global Environment (CHanGE), Medic undertook this scoping review to inform the design and implementation of digital tools that can better help frontline workers identify, mitigate, and respond to climate-sensitive health outcomes. We identify and review literature centered on the impacts of climate-related stressors on healthcare service delivery by community health workers (CHWs), with a focus on climate-sensitive health outcomes in these countries. The review explored existing digital tools, including the use of artificial intelligence (AI), and its application in reducing and adapting to climate-sensitive health outcomes. By scrutinizing the current landscape of technological interventions, the review aims to shed light on potential gaps, offering insights into areas where advancements in AI and related technologies can be harnessed to enhance the resilience of community health services and the adaptive capacity of health systems in these countries. Ultimately, the objective is to contribute knowledge that can guide future developments and interventions, ensuring that technologies might better align with the specific needs and challenges posed by climate-related stressors in the context of healthcare delivery.

## 2 Methods

This scoping review employed the five-stage framework proposed by Arksey & O’Malley [10] to identify, select, and synthesize relevant literature on vulnerability factors, exposure pathways, and interventions in geographic regions where Medic has existing CHT deployments.^1^

The focus was on published literature that explored the deployment of AI as a potential tool for community health systems to reduce and adapt to climate-sensitive health outcomes.

Specifically, this review aims to answer the following research questions:

1. What are key vulnerability factors in regions where Medic has a presence?
2. What exposure pathways result in climate-sensitive health risks in the geographies covered?
3. What are the potential pathways through which climate-related stressors affect health care delivery by community health workers?
4. What community-level and health system interventions or adaptations are documented to reduce climate-sensitive health risks?
5. What technologies, such as Artificial Intelligence, are being used to inform health community systems to help reduce the impacts of climate-sensitive health risks?

The search strategy examined peer-reviewed academic literature considering studies published in English since 2000. Table 1 illustrates the search terms, while the queries used to extract relevant articles from diverse databases, including PubMed, Google Scholar, Scopus, and Web of Science are provided in (Supplementary Material 1). Additionally, an exploration of gray literature was undertaken, focusing on reports and case studies disseminated by development partners such as World Health Organization (WHO), United Nations Development Programme (UNDP), the World Bank Group, and the United States Agency for International Development (USAID).

**Table 1:** Scoping review search terms.

Climate change terms	Climate-sensitive health outcomes	Health professionals	Intervention related terms	Artificial intelligence related terms
Climate Climate change Climate variability Climate warming Extreme weather Heat waves Climatic hazards Drought Flooding	Airborne diseases/ Meningitis Vector borne diseases/ malaria/ yellow fever/ dengue fever/ Rift Valley fever Waterborne diseases/ cholera/ diarrheal diseases/ enteric diseases	Health professionals Community Health Workers Healthcare providers Doctors/ physicians Primary healthcare providers General practitioner	Mitigation Adaptation Adaptive capacity Adaptation measure Interventions Preparedness Surveillance	Artificial Intelligence Predictive models Digital technologies Forecasting Predictive analytics Machine learning Neural networks

The inclusion of studies in this scoping review involved a two-stage process conducted by two reviewers. Initially, a screening phase was conducted by a single reviewer (SS), where published studies were assessed based on their titles and abstracts. This preliminary evaluation aimed to identify studies that held potential relevance to the overarching research questions. Subsequently, articles meeting the criteria were subjected to a more detailed review in the second stage by SS under the supervision of two reviewers CA and DC.

Selection for inclusion in the full review was contingent upon satisfying one or more of the following inclusion criteria:

1. Identified specific climate-related stressors (e.g. floods, droughts), quantified their impact on health system infrastructure (e.g., damage to facilities, disruption of supply chains), and described effects on healthcare professionals (e.g., increased workload, heat stress, mental health).
2. Detailed specific impacts of climate change on healthcare service delivery and described its effects on community health workers' performance.
3. Outlined proposal or evaluation of specific, low-cost, scalable interventions (e.g., addressing infrastructure resilience, workforce capacity building, or service delivery adaptation) for climate resilience in contexts relevant to Medic's operations.
4. Used Artificial Intelligence, machine learning, or specific digital tools for climate-health predictions or interventions, providing evidence of their effectiveness or potential impact.

A data extraction tool in Microsoft Excel (Supplementary Material 2) was developed and implemented throughout the review to facilitate data collection and ensure consistency in information retrieval. The tool was designed to capture published primary data that would provide evidence on specific themes to inform the research questions. The categories were *(i)* climate-related stressors and health exposure pathways in countries where Medic operates, *(ii)* climate-related stressors and health professionals, and *(iii)* the use of technologies to predict and adapt to the impacts of climate-related stressors on healthcare. This was further broken down to emphasize *(iv)* the use of AI in climate-related stressors and health research.

The data extraction tool served as a systematic guide, enabling extraction of information relevant to the objectives. The tool’s specific components included the study’s geographic region, climate-sensitive health outcomes addressed, and subcategory of climate-sensitive diseases investigated, characteristics of the study population, methods employed, key findings, temporal considerations, and relevant references. Additionally, it included comments that provided further context on methods or results and clarified any abbreviations. The findings were collated, analyzed, and summarized to answer the research questions. Initial categorization of the reviewed studies was based on thematic areas corresponding with the research questions and the data extraction tool. The review’s results were distributed by geographic region to allow for illustrative comparison between the contextually different geographic areas.

Results were also organized by exposure pathways to examine the specific mechanisms through which climate change impacts health outcomes. Finally, the results were distributed by research methods to provide insights into the diversity of approaches utilized.

## 3 Results

### 3.1 Study identification, screening, and eligibility

The database search generated 3184 studies, of which 3084 were excluded based on the article title. Abstracts of the remaining 96 studies were then reviewed, resulting in exclusion of 29 studies with inelligible article type or geographic region, and another 41 studies which ultimately didn’t meet the inclusion criteria. A total of 30 studies were selected for the review, including 4 reports and articles from gray literature. The study selection flow chart is presented in Fig. 1.

**Studies retrieved through search in several databases (n = 3,180)Articles identified from gray literature (n=4)Studies excluded by title (n=3,084)Studies excluded by abstract (n=29)Studies excluded based on inclusion criteria(n=41)Total articles included in the review (n = 30)Studies screened for abstract(n=96)Articles assessed for eligibility (n=67)Articles included in the review (n=26)IdentificationScreeningEligibilityIncludedStudies retrieved through search in several databases (n = 3,180)Articles identified from gray literature (n=4)Studies excluded by title (n=3,084)Studies excluded by abstract (n=29)Studies excluded based on inclusion criteria(n=41)Total articles included in the review (n = 30)Studies screened for abstract(n=96)Articles assessed for eligibility (n=67)Articles included in the review (n=26)IdentificationScreeningEligibilityIncludedFig. 1:**
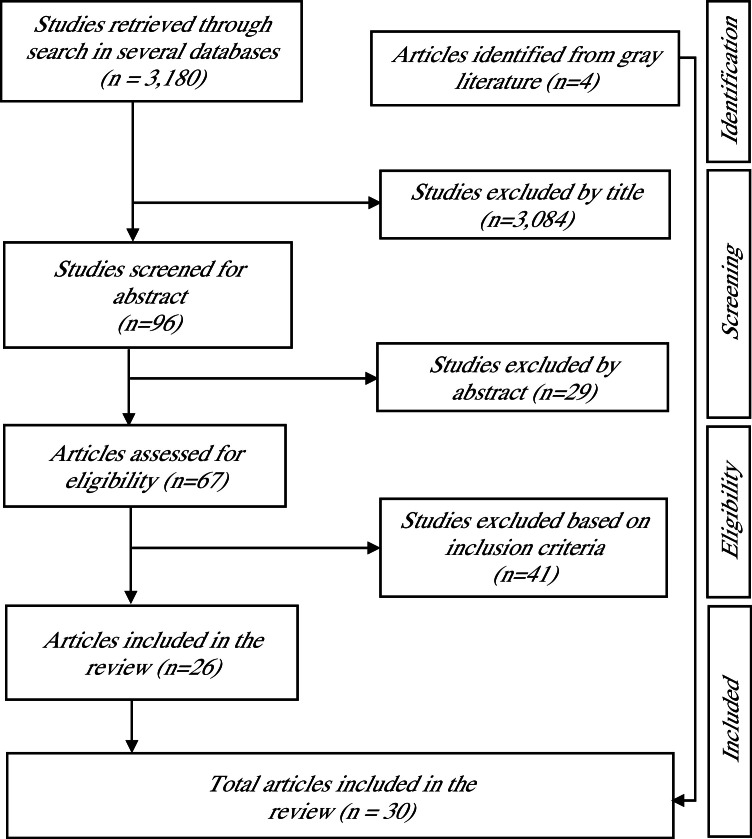
Flowchart of study selection process.

### 3.2 Description of studies

#### 3.2.1 Distribution by region and climate-sensitive health outcomes

The review focused on studies in geographic locations where Medic operates, with a significant number of studies from India and South Africa (*n* = 10), fewer studies from Nepal and the Philippines (*n* = 3), and most studies from the African region (*n* = 15) distributed across countries, including Tanzania, Uganda, Mali, Nigeria, and Burkina Faso. Most studies focused on waterborne diseases (*n* = 18) across the African region specifically, while vector borne diseases were prevalent across all regions (*n* = 8), noting that studies from India, Nepal, and Philippines particularly focused on malaria and dengue fever. Studies that focused on food security emerged from African countries (*n* = 7). Furthermore, studies that addressed extreme events emphasized extreme heat and floods (*n* = 8). Few studies were identified that addressed airborne diseases (*n* = 3) with one study focusing on meningitis in Niger. Additionally, one case study was identified in the gray literature that assessed mental health in relation to climate change from different African countries.

#### 3.2.2 Distribution by research methodology

The reviewed studies used a variety of research methods, only considering studies with primary data. Four studies used a combination of qualitative research methods. Many studies utilized mathematical modeling and AI tools (*n* = 12), while only one study used thematic content analysis and one study used a case study analysis.

### 3.3 Thematic analysis of the studies

#### 3.3.1 Climate change and health exposure pathways

Vector borne diseases were the major exposure pathway in this category, particularly dengue fever, malaria, and rift valley fever [[11], [12], [13]]. Studies (*n* = 9) emphasized the threat of droughts and changing rain patterns on water security, leading to reduced high-quality food crop production and increased malnutrition [14]. Additionally, increasing temperatures contributed to the prevalence of diseases such as meningitis [15].

#### 3.3.2 Climate change and health professionals

Studies (*n* = 3) that assessed the perspectives of health professionals in the context of health systems interventions and climate action focused mainly on exploring the impact of climate change on health in the sampled regions. They touched on the damage to healthcare facilities by weather-related disasters, lack of resources to cope with infectious disease outbreaks, and the rise of waterborne diseases from excessive rainfall and floods [16]. Moreover, studies emphasized the stress and isolation experienced by healthcare workers in remote areas, driving brain drain and health center closures. Urban-rural migration also contributed to vulnerability, resulting in overcrowding, poor sanitation, and an increased risk of diarrheal diseases. However, there was also a notable lack of awareness among healthcare workers regarding climate change adaptation measures [17]. In one study, most health professional participants acknowledged the reality of climate change, with waterborne diseases, malnutrition, respiratory illnesses, and mental health severely affected [18].

#### 3.3.3 Use of technologies to predict and adapt to the impacts of climate change on healthcare

Most of the studies (*n* = 7) in this category focused on the use of mathematical regression models and the Climate Suitability Malaria Transmission (CSMT) Model to provide useful information for health systems adaptation to climate change. The CSMT Model is a tool designed to predict geographical areas where the climate is suitable for malaria transmission. Initially focusing on temperature, rainfall, and humidity [19], the model evolved over the years to incorporate more complex environmental factors. It provides a scientific basis for projecting how climatic factors influence malaria transmission [20]. This, in turn, informs strategies for public health interventions, particularly in regions most vulnerable to climate change.

A Poisson regression model to confirmed that high incidences of dengue were linked to post-monsoon months and projected an increase in future cases, particularly from September to November and December to February, due to favorable climatic conditions [21]. A study projected the extension of malaria transmission windows, with the spatial extent of transmission windows increasing by the 2030s for *Plasmodium vivax* (Pv) and *Plasmodium falciparum* (Pf). A GIS-based model projected the expansion of high-risk zones for malaria, particularly in the northern and eastern parts of Burundi, due to changes in weather variables [22]. A linear regression analysis projected the expansion of malnutrition in relation to the shift in rain patterns across Mali [23]. A study highlighted the critical need for climate change adaptation in the context of health and community resilience, emphasizing the importance of expanding the roles of Community Health Workers beyond maternal care and into climate action and suggesting the integration of climate action into primary health care interventions to strengthen communities’ adaptive capacity to climatic hazards [24].

#### 3.3.4 The use of AI in health adaptation to climate change

Studies (*n* = 5) focusing on AI highlighted current deployments to address climate-related challenges, providing a comprehensive overview of the use of AI tools in forecasting disease outbreaks, particularly dengue [25], diarrhea, and cholera, and the potential impact of climate change on their distribution and transmission. These tools also identify the climatic variables directly associated with these outbreaks that can be used for modeling. Studies compared different Machine Learning (ML) models and neural networks and highlighted their strengths, whether in prediction capacity or in managing unbalanced data [26,27].

Collectively, the studies emphasized the effectiveness of different ML methods in outbreak prediction, and the potential of deep learning methods, such as Long Short-Term Memory (LSTM) and Convolutional Neural Network (CNN), in improving the accuracy of outbreak forecasts. One study used such models to identify mean temperature as a variable of importance for dengue distribution, and to project the increase in dengue incidence under different climatic scenarios [25]. Such models drive targeted vector control measures such as mosquito spraying or habitat management, reducing disease transmission, and protecting community health.

## 4 Discussion

The scoping review identified several exposure pathways through which climate-related stressors impact individual, community, and population health in the sampled geographic regions.

The results are consistent with published literature on the greater vulnerability of low- and middle-income countries (LMICs) to climate change impacts on human health. Falling in line with the IPCC’s 6th Assessment Report, it highlighted how the largest adverse impacts of climate-related stressors and climate extreme events on food and water security have been observed in many locations and communities in Africa, Asia, and Least Developed Countries (LDCs) [28].

Findings showcased direct and indirect negative impacts of climate-related stressors on health systems and health professionals in particular, drawing further attention to the need for health professionals to adopt new roles and responsibilities in responding to climate-related health risks [29]. Despite the growing knowledge among health professionals on the threat climate-related stressors pose on health systems, significant gaps remain in mainstreaming climate-related stressors in the education and training of health professionals, particularly in LMICs compared to High-Income Countries (HICs) [30]. Some successful case studies of community healthcare adaptations to climate-related stressors focused on building the capacity of Community Healthcare Workers (CHWs) to identify and tackle climate-related health risks and promote resilient communities [31]. However, the search results revealed limitations in finding published literature on adaptation interventions in community health, and healthcare delivery more broadly, that employ AI. This gap may be attributed to the novelty and evolving nature of AI applications in healthcare, and the need for more research and real-world implementation to generate substantial evidence.

The exploration of the role played by advanced technologies in forecasting and preparing for climate-related risks sheds light on the true potential of ML models and AI in supporting healthcare adaptation. While AI has the potential to increase the resilience of healthcare systems to the health risks of climate-related stressors, its integration into healthcare delivery, and adaptation interventions, particularly on the community level, remains largely underexplored. For example, AI might support such community-level interventions that help frontline workers identify and assess vulnerabilities while prioritizing those at the forefront of community health systems to anticipate and respond to climate-sensitive health risks. This might be: (*i*) through an adaptive climate resilience toolkit designed for CHWs, which provides a menu of options to help them link and navigate current and shifting exposure pathways with health outcomes; (*ii*) an early warning system to predict climate related disease burdens; (*iii*) or, in facilitating the analyses of vast volumes of climate and health related data to inform community health system policy.

## 5 Strengths and limitations

Considering the multiple databases searched, as well as gray literature, along with the extensive and inclusive search terminology, we believe we identified a majority of the peer-reviewed, published literature on the impacts of climate change on community healthcare service delivery, with a focus on climate-sensitive health outcomes in low and middle-income countries (LMICs).

Relevant literature published solely in other languages, such as Hindi, Nepali, Arabic, Spanish or French was missed. Another limitation is that we did not perform a quality assessment of all articles included in the review [32], which may result in lower quality studies included in the review and results.

## 6 Conclusion

Our review identified critical gaps in the literature on community-level health interventions to reduce climate-sensitive health risks. Overall, the current literature is skewed towards community perception of climate-sensitive health outcomes and adaptation challenges faced by the health system [14]. There is a need for future interventions, and studies of these interventions, that can shed future light on the intersection of climate variability and interventions aimed at addressing climate-related health outcomes.

## Funding

The research, writing and publication costs were funded by Medic.

## Disclaimer

The views and opinions expressed herein do not reflect the official views or policies of affiliated institutions.

## Ethical consideration

The study did not need ethical approval as it did not have direct contact with human subjects.

## CRediT authorship contribution statement

**Sonyta Saad:** Writing – review & editing, Writing – original draft, Validation, Methodology, Investigation, Formal analysis, Data curation. **Chrisgone Adede:** Writing – review & editing, Validation, Supervision, Methodology, Conceptualization. **David Citrin:** Writing – review & editing, Writing – original draft, Validation, Supervision, Methodology. **Beatrice Wasunna:** Writing – review & editing, Validation, Supervision, Methodology. **Mourice Barasa:** Writing – review & editing, Visualization, Validation, Methodology. **Krishna Jafa:** Writing – review & editing, Supervision, Methodology, Funding acquisition. **Kristie L. Ebi:** Writing – review & editing, Validation, Supervision, Methodology.

## Declaration of competing interest

The authors declare that they have no known competing financial interests or personal relationships that could have appeared to influence the work reported in this paper.

## Appendix Supplementary materials

Image, application 1

Image, application 2

## Footnotes

1 Burundi, Democratic Republic of Congo, Ghana, India, Kenya, Malawi, Mali, Nepal, Niger, Philippines, Republic of South Africa, Togo, Uganda, Zanzibar (Tanzania), and Zimbabwe.
