# Supplementary material for: Climate-related stressors, community healthcare systems, and adaptation strategies: A scoping review
Source: J Clim Chang Health. 2025 Nov 5;26:100574. doi: 10.1016/j.joclim.2025.100574 (PMC12851238; doi:10.1016/j.joclim.2025.100574)
Supplement: Supplementary file 1 [file mmc1.docx]

**Supplementary Material 1: PubMed query examples**

| **Concept No.** | **Search Concept** | **Sub-Concepts** | **Example Search Terms** |
| --- | --- | --- | --- |
| **1** | ***Exposure pathways*** | Air-borne diseases | ((Climate change[Title/Abstract]) OR (Climate variability[Title/Abstract]) OR (Climate variation[Title/Abstract]) OR (Climate warming[Title/Abstract]) OR (Climatic hazards[Title/Abstract]) OR (Climate[Title/Abstract]) OR (Heat waves[Title/Abstract]) OR (Extreme weather[Title/Abstract]) OR (Drought[Title/Abstract]) OR (Flooding[Title/Abstract])) AND ((Vector borne diseases [Title/Abstract]) OR (Vector-borne diseases[Title/Abstract])OR (Malaria[Title/Abstract]) OR (Dengue fever[Title/Abstract]) OR (yellow fever[Title/Abstract]) OR (Rift Valley fever[Title/Abstract]) OR (Water borne diseases[Title/Abstract]) OR (Food borne diseases[Title/Abstract]) OR (Food-borne diseases[Title/Abstract]) OR (Cholera[Title/Abstract]) OR (Non-cholera Vibrio spp[Title/Abstract]) OR (Human Enteric diseases[Title/Abstract]) OR (diarrheal diseases[Title/Abstract]) OR (Airborne diseases[Title/Abstract]) OR (Meningococcal meningitis[Title/Abstract])) AND (("2000/01/01"[Date - Publication]:"2023/09/30"[Date - Publication])) |
|  |  | Water-borne diseases |  |
|  |  | Vector-borne diseases |  |
|  |  | Food-borne |  |
| **2** | ***Community health systems & Health professionals*** | Health Professionals | ((Climate change[Title/Abstract]) OR (Climate variability[Title/Abstract]) OR  (Climate variation[Title/Abstract]) OR (Climate warming[Title/Abstract]) OR  (Climatic hazards[Title/Abstract]) OR (Climate[Title/Abstract]) OR  (Heat waves[Title/Abstract]) OR (Extreme weather[Title/Abstract]) OR  (Drought[Title/Abstract]) OR (Flooding[Title/Abstract]))  AND  ((Community health workers[Title/Abstract]) OR (Health professionals[Title/Abstract]) OR (Doctors[Title/Abstract]) OR  (Physician[Title/Abstract]) OR (Healthcare worker[Title/Abstract]) OR  (primary health care provider[Title/Abstract]) OR (General practitioner[Title/Abstract]) OR (Health worker[Title/Abstract]) OR  (Health care professional[Title/Abstract]))  AND  ((Migration[Title/Abstract]) OR (Mitigation[Title/Abstract]) OR  (Adaptation[Title/Abstract]) OR (Adaptation measure[Title/Abstract]) OR  (Intervention[Title/Abstract]) OR (Preparedness[Title/Abstract]) OR  (Adaptive capacity[Title/Abstract]) OR (Surveillance[Title/Abstract]) OR  (Artificial intelligence[Title/Abstract]) OR (Machine learning[Title/Abstract]) OR  (Digital technologies[Title/Abstract]) OR (Forecasting[Title/Abstract]) OR  (Predictive analytics[Title/Abstract]) OR (Expert systems[Title/Abstract]) OR  (Knowledge engineering[Title/Abstract]) OR (Neural networks[Title/Abstract]))  AND (("2000/01/01"[Date - Publication]:"2023/09/30"[Date - Publication])) |
|  |  | Community healthcare workers |  |
|  |  | Community healthcare systems |  |
|  |  | Adaptation Strategies |  |
|  |  | Digital Technologies |  |
| **3** | ***Digital tools & health systems adaptation/mitigation*** | Machine Learning | ((Climate change[Title/Abstract]) OR (Climate variability[Title/Abstract]) OR  (Climate variation[Title/Abstract]) OR (Climate warming[Title/Abstract]) OR  (Climatic hazards[Title/Abstract]) OR (Climate[Title/Abstract]) OR  (Heat waves[Title/Abstract]) OR (Extreme weather[Title/Abstract]) OR  (Drought[Title/Abstract]) OR (Flooding[Title/Abstract]))  AND  ((Artificial intelligence[Title/Abstract]) OR (Machine learning[Title/Abstract]) OR  (Digital technologies[Title/Abstract]) OR (Forecasting[Title/Abstract]) OR  (Predictive analytics[Title/Abstract]) OR (Expert systems[Title/Abstract]) OR  (Knowledge engineering[Title/Abstract]) OR (Natural language processing[Title/Abstract]) OR  (Neural networks[Title/Abstract]) OR (Robotics[Title/Abstract]))  AND  ((Mitigation[Title/Abstract]) OR (Adaptation[Title/Abstract]) OR  (Adaptation measure[Title/Abstract]) OR (Intervention[Title/Abstract]) OR  (Preparedness[Title/Abstract]) OR (Adaptive capacity[Title/Abstract]) OR  (Surveillance[Title/Abstract]))  AND (("2000/01/01"[Date - Publication]:"2023/09/30"[Date - Publication])) |
|  |  | AI |  |
|  |  | Digital Technologies |  |
|  |  | Adaptation/mitigation |  |
