# Supplementary material for: Climate-related stressors, community healthcare systems, and adaptation strategies: A scoping review
Source: J Clim Chang Health. 2025 Nov 5;26:100574. doi: 10.1016/j.joclim.2025.100574 (PMC12851238; doi:10.1016/j.joclim.2025.100574)
Supplement: Supplementary file 2 [file mmc2.docx]

**Supplementary Material 2: A summary of studies included in the study**

Table 1: A summary of the studies included in the scoping review

| **Region** | **Climate-sensitive health outcomes** | **Sub-category of climate-sensitive: diseases** | **Study population** | **Methods** | **Temporal scale** | **Results** | **Reference** |
| --- | --- | --- | --- | --- | --- | --- | --- |
| West Africa, South Africa, East Africa (Ghana, Nigeria, South Africa, Namibia, Ethiopia, and Kenya) | vector-borne diseases/ water-borne diseases | Malaria/ cholera | Healthcare professionals 122 respondents | cross-sectional study | 2019 | 63.1% of the total respondents reported that climate change had been extensively experienced in the past years, while 32% claimed that the sampled countries had experienced them to some extent. The sampled African countries face a significant challenge to infections caused by climate change impacts. | Opoku et al, 2021 |
|  |  |  |  |  |  | Nigerian respondents recorded the highest levels (67.7%), followed by Kenya with 66.6%. South Africa had the lowest level of impact as perceived by the respondents (50.0%) when compared with the other sampled countries. All respondents from Ghana and Namibia reported that health problems caused by climate change are common in the two countries. |  |
|  |  |  |  |  |  | As perceived by the health professionals, the inadequate resources reiterate the need for infrastructural resources, medical equipment, emergency response resources, and technical support. |  |
|  |  |  |  |  |  | Respondents from West Africa (Ghana, Nigeria) reported that floods and extreme temperatures are frequent, and those from Eastern Africa (Ethiopia, Kenya) mentioned drought and extreme temperatures. Extreme temperatures appear to be common in the Western and Eastern part of Africa. The Southern African countries (South African and Namibia) experienced much drought and extreme temperatures. To some extent, floods and erratic rainfall pattern were observed in all the sampled countries. |  |
| Tanzania | water-borne diseases | Cholera | 2951 patients | design science research methodology/ supervised machine learning algorithms | 2017 - 2019 | XGBoost and K- NN perform best with respect to the chosen metrics (specificity/ sensitivity/ accuracy) | Leo et al, 2019 |
|  |  |  |  |  |  | XGBoost has great applicability when data is imbalanced |  |
|  |  |  |  |  |  | data analysis indicated that there was a greater load of cholera cases during August/ September and April |  |
| Coastal India | water-borne diseases | Cholera | 40 districts | Random Forest model/Decision Tree/a Neural Network (Multi-Layer Perceptron) | 2010–2018 | The developed Random Forest model predicted 89.5% of cholera outbreaks | Campbell et al, 2020 |
|  |  |  |  |  |  | The pre-monsoon (March, April, May) outbreak made up 42.1% of all outbreaks with the highest sensitivity score (0.933) |  |
|  |  |  |  |  |  | The model successfully predicted outbreaks on the district-level |  |
|  |  |  |  |  |  | the RF model performed better than the multyi-layer perception neural network and the decision tree |  |
|  |  |  |  |  |  | usage of Essetial climate variables (ECVs) for cholera-outbreak risk analysis: soil moisture contributed more strongly than precipitation towards improving the model performance. Sea surface temperature has been found to influence cholera variability from warming waters increasing moisture convergence and subsequently precipitation. |  |
|  |  |  |  |  |  | chlorophyll-a concentration, sea surface salinity and land surface temperature are the strongest predictors of the cholera outbreaks in the dataset used |  |
| South Africa |  | Diarrhoeal Diseases | 9 provinces (Western Cape, Eastern Cape, Northern Cape, North West, Free State, Limpopo, KwaZulu Natal, Gauteng, and Mpu- malanga) | Machine learning methods | 2008 - 2019 | the most influential climate variables to be considered when predicting diarrhoeal outbreak are precipitation/ humidity/ evaporation /temperature conditions. | Abdullahi et al, 2022 |
|  |  |  |  | Deep learning methods: Convolutional Neural Networks (CNNs) and Long-Short term Memory Networks (LSTMs) |  | the prediction capacity of the DL methods CNN was found to be superior (with statistical significance) in terms of prediction accuracy across most provinces |  |
|  |  |  |  | Support Vector Machine (SVM) |  | the amount of training data used for training, significantly affects the prediction performance of all the three ML methods, because the predictions made with the augmented datasets yielded better and lower (RMSE) than their predictions with the real-world data-sets |  |
|  |  |  |  |  |  | CNN outperformed the other methods when the real-world dataset was used alone while LSTM outperformed the other methods when either of the augmented datasets were used |  |
|  |  |  |  |  |  | The sensitivity study showed that the relative importance of each climate variable differs across provinces when training any given diarrhoea outbreak prediction method |  |
|  |  |  |  |  |  | Results showed that deep neural networks outperform traditional ML algorithms for most disease prediction tasks |  |
| India | Vector-borne diseases | Dengue fever | Kerala state (59,801 total reported dengue cases during the temporal scale) | Statistical method vector auto regression (VAR), machine learning methods support vector regression (SVR) and (GBM), and deep learning method (LSTM) | 2003 - 2017 | The models captured the trends of the dengue cases but only the LSTM and SVR models successfully predicted the outbreak periods | Kakarla et al, 2022 |
|  |  |  |  |  |  | the Generalized boosted regression model (GBM) showed that relative humidity (RH) is the highly influencing variable whereas mean temperature is the least influencing variable on dengue transmission and that each climate variable is influencing dengue prevalence at different levels. |  |
|  |  |  |  |  |  | The Support vector regression (SVR) has given a high priority for rainfall and least priority for mean temperature |  |
|  |  |  |  |  |  | The long short-term memory deep learning method (LSTM) model outperformed other three models. The predicted results of LSTM are quite similar with the real case scenarios of Kerala |  |
|  |  |  |  |  |  | During the correlation analysis between dengue cases and weather variables, rainfall, soil moisture, relative humidity, and Nino3.4 showed positive correlations, while mean temperature showed negative cor- relation with dengue |  |
| Nepal | Vector-borne diseases | Dengue fever | 124 dengue presence locations of reported disease cases | MaxEnt modeling using the dengue presence locations and 3 bioclimatic variables as predictor variables. The Community Climate System Model (CCSM) was used to model three future greenhouse gas concentration trajectories and grid-based population data was used to approximate the affected human population. | 2050s and 2070s | The MaxEnt model’s Jackknife test of variable importance showed that the mean temperature of the wettest quarter was the variable with the highest gain when used in isolation. Indicating it holds the most information for dengue distribution among the variables used for model development. About 24.43% area of the country is presently climatically suitable for dengue fever. The model depicts that the climatically suitable area of dengue fever would increase in the 2050s and 2070s under all the RCPs. The total climatically suitable areas would increase under the very high emission scenario (RCP8.5) compared to other scenarios. In 2070 the proportion of highly suitable areas would increase under the RCP 2.6 emission scenario. The proportion of the population exposed to dengue fever would increase under all of the emission scenarios. The southern low lands of the country are suitable for dengue fever and for other mosquito-borned diseases (Malaria, chikungunya, encephalitis). | Acharya et al, 2018 |
|  |  |  |  |  |  |  |  |
| Nepal | Vector-borne diseases | Malaria | 964709(Morang district) 775,709(Kailali district) | Retrospective study, Generalized additive mixed models (GAMM) were used to assess the effects of climatic variables on malaria incidence using the district-level monthly aggregated data | 2004-2012 | Among climatic variables, only minimum temperature and relative humidity were significant predictors of malaria incidence. Overall, a 1°C increase in temperature increased malaria incidence by 25% | Dhimal et al, 2014 |
| Metro Manila, Phillippines | Vector-borne diseases | Dengue fever | Metro Manila population (monthly dengue incidence data) | Retrospective study, the monthly dengue incidence data, and monthly climatice factors (mean temperature and mean rainfall) data were used and linked through a multiple linear regression analysis. The model assumed that dengue incidence is a function of temperature and rainfall measurements. A stepwise regression procedure was used in building the model. | 1996 - 2005 | The study found that the highest incidence of dengue fever was in September 1998, significant difference was observed for the monthly rainfall, but no significant correlation was found between temperature and dengue incidence. Indicating that only rainfall plays an important role in the occurrence of dengue in Metro Manila. | Sia Su et al, 2008 |
| Africa | Vector-borne diseases | Rift valley fever | Rift valley fever incidence cases were collected from (Botswana, Kenya, Madagascar, Mauritania, Namibia, Senegal, South Africa, Sudan and Swaziland, with by far the majority (90%) coming from South Africa) | Bayesian spatial model for rift valley fever occurrence across Africa, along with spatial environmental (bioclimatic and rain events variables) and habitat data. RVF cases were modelled within 4 periods across the year, modelled within 3 climatic oscillation groups, and then a nested structure of 12 possible states by combining these four seasonal and three climatic oscillation groupings was created, these groups were modeled simultaneously. | 2017 | Across the 4 seasonal periods, January-June contained the majority of all cases (82%), with overall RVF cases higher in the years with strong El Niño or La Niña oscillations. In the case of strong El Niño event years, the majority of cases were recorded in South Africa and Kenya. In the case of strong La Niña, cases were predominately recorded in South Africa, but also in North African countries. | Redding et al, 2017 |
|  |  |  |  |  |  | The study found that the risk of RVF increased with the presence of irrigation, a larger proportion of land under cultivation, and a higher human population density. |  |
|  |  |  |  |  |  | The spatial prediction of RVF found that years with no designated climatic oscillations show patterns of low risk in quarters 1 and 2 (January–June), but high and wide-spread risk in western Africa, but for both strong El Nino and La Nina event years quarters 1 and 2 have many high-risk areas, including the endemic areas. For strong El Nino event years, quarter 1 had especially high-risk areas, as expected, especially in South Africa, and in Kenya, Tanzania, and Uganda |  |
| Sub-saharan Africa | water and vector-borne diseases | Malaria, cholera, diarrheal diseases | 30 participants in Focused Group Discussions | Exploratory qualitative study | 2019 | Participants mentioned how droughts and changing rain patterns threaten water security which reduces high-quality food crop production induces malnutrition. Increasing temperatures leads to the prevalence of Malaria and meningitis. | Scheerens et al, 2021 |
|  |  |  |  |  |  | Healthcare facilities are damaged by climatic disasters |  |
|  |  |  |  |  |  | health facilities lack resources to cope with infectious diseases outbreaks |  |
|  |  |  |  |  |  | Participants identified water-borne diseases to rise from excessive rainfall, floods and cyclones |  |
|  |  |  |  |  |  | Participants mentioned how healthcare workers in remote and affected areas experience stress and feel unsupported and isolated (leading to brain drain and health centres closures) |  |
|  |  |  |  |  |  | urban-rural migration can result in overcrowding and dependence on scarce resources (water shortages) which can lead to poor sanitation and diarrheal diseases |  |
| Kolkata, India | Vector-borne diseases | Dengue fever |  | The study developed zero-inflated Poisson regression model, dengue cases were framed as the response variables, while the meteorological factors (maximum temperature, minimum temperature, relative humidity, rainfall) as explanatory variables. | 2020 - 2100 | High incidences of dengue were found in post-monsoon months when the temperature, humidity and rainfall are ideal for the dengue virus to multiply. It is estimated for 81 years (2020–2100) that future dengue cases during September to November will always be higher, with a higher number in RCP8.5. During December to February, increase in CO2 concentrations under RCP8.5 leads to suitable warmer weather situations for vector survival and multiplication and predict more than twice as many dengue cases than RCP4.5, this could be due to the global warming during the winter months in the RCP8.5 scenario. | Bal et al, 2020 |
| India | Vector-borne diseases | Malaria |  | The study developed a (FCSMT) model under Geographical Information Sysytem (GIS) environment, focusing on P.vivax and P. facliparum as the main malaria parasites. Climate data on temperature and relative humidity were extracted from CORDEX South Asia, and malaria epidemiologic data (2013 -2017) were obtained, and the most suitable range was selected for defining temperature and RH suitability thresholds. The model inlcuded 6 stages: generation of monthly interpolated temperature and RH maps for both periods, determination of fuzzy membership functions for both indices (temperature and humidity), creation of fuzzy-monthly temperature and RH suitability maps, month-wise generation of climate suitability maps for both periods, generation of transmission-windows-based climate suitability map for both periods, generation of climate suitability change map. | Baseline 1967- 2005 future projection 2021 - 2040 | Monthly climatic suitability maps (combining temperature and RH) for Pv and Pf. show that the northern half of India remains least suitable for malaria transmission in both periods. The transmission suitability improves with the monsoon onset, with 80% of the country becoming suitable at different scales. With all the projected outcomes indicating an extension in the malaria transmission windows. | Sarkar et al, 2019 |
|  |  |  |  |  |  | The generated composite climate suitability maps for malaria transmission shows that the spatial extent of TWs of 4–6 months and 10–12 months are projected to increase by 2030s for both pv and pf. Eastern coastal belt, mainly Odisha and West Bengal, are projected to experience increased TWs for Pv malaria transmission by the 2030s. |  |
|  |  |  |  |  |  | The climate suitability change map shows the changes in number of months of transmission windows (new foci/extension/reduction) projected to occur by the 2030s as compared to baseline years. In the western Himalayan states, some areas are likely to have new foci of transmission, while other areas are more likely to have a spatial extension of TWs by two months. Some coastal areas will likely become suitable for pf malaria transmission, while others are projected to experience reduction in number of months of transmission windows. |  |
| Uganda, Kanungu District | Vector-borne diseases, airborne diseases and gastrointestinal diseases | Malaria, acute respiratory infections, and gastrointentinal diseases | hospital admissions of the 252,000 residents | A mixed-effects Poisson regression model was used to investigate the association between hospital admissions and season, precipitation, and temperature. Two multivariable linear regressions were used to examine the effect of season and year on both climatic variables (average daily temperature and total daily precipitation). In th model, the outcome variable was number of hospital admissions per day, and the exposure variables were meteorological paramteres. Descriptive statistics were used to describe admission patterns, with admission data collected from records, and they were used to examine indpendent variables. Climatic data (daily values of total precipitation (rainfall), maximum, minimum, and average temperature) were collected from (ERA)-Interim Climate Database. | 2011 - 2014 | The most common diagnoses were acute respiratory infections, malaria, and acute gastrointestinal illness. Average daily temperature increased annually, while average daily total rainfall showed no significant trend over time. The model showed that admissions were higher during the dry season, compared to the rainy season. When controlling for confounders, the admission rate for all diagnoses were significantly higher during when the average temperature was extremely high. | Bishop-Williams et al, 2018 |
| Uganda, Kanungu District | Vector-borne diseases, food security, airborne diseases, gastrointestinal diseases | Malaria, malnutrition, stomach disorders, and respiratory disease | 6700 Batwa communities (Mukongoro and Kihembe) | Qualitative research that employed 5 Rapid Rurual Appraisal techniques (Semi-structured interviews with households, Key informant interviews, future storylines, Biographies, and PhotoVoice) to identify key climate-sensitive, community-identified health outcomes, describe determinants of sensitivity at multiple scales, and characterize adaptive capacity of Batwa health systems. | 2010 | Climate-sensitive health outcomes: 4 of the 5 major health problems identified by the community were climate-sensitive health outcomes(malaria, malnutrition, stomach disorders, and respiratory disease). Households identified malaria as a major concern, with incident being more common at the beginning of both the dry and the rainy seasons, due to stagnant water, followed by malnutrition. Exposure pathways: water, as poluuted water sources and insufficient amounts. Food security in both quality and quantity. Vector-borne diseases, with significant breeding opportunities. Extreme weather events. | Berrang-Ford et al, 2012 |
|  |  |  |  |  |  | The study identified human drivers of sensitivity on 3 levels. On the individual level, children and pregnant women are most sensitive. On the community level, low socioeconomic status, social and political marginaltization, and livelihood transition were identified. On the regional level, demorgaphics, economy and conflict were identified. |  |
|  |  |  |  |  |  | In assessing the adaptive capacity of the community, the study found that the communities relied on traditional medicine as a first course of treatment before seeking Western healthcare. Community memebers reported problems in accessing Western healthcare due to financial cost and discrimination, with dependence on facilities operated by development organizations. Informal institutions that contribute to the communities' adaptve capacity was identified as community support networks, which were exhibited at different degrees within the communities. |  |
| Burundi | Vector-borne diseases | Malaria |  | The study used a GIS-based model to map malaria risk as a function of climatic and topographic conditions. Climatic variables used were temperature, rainfall and relative humidity, and they were obtained under the RCP8.5 scenario. Data on malaria incidence was obtained based on cases reported in Burundi, and Weighted Overlay analysis was applied to generate a new raster surface representing different levels of malaria risk according to the considered climatic and topographic determinants. Overlay Weighted analysis was also used to map malaria risk under RCP2.6 and RCP8.5 through future climatic data and topographic data. Both risk maps were compared to identify the trend and scale of malaria risk under climate change up to 2050. | 2040 -2050 | The study produced a risk map in which it classified areas into three levels, low, moderate and high malaria risk. Based on current climatic conditions, major proportions of Burundi land surface have moderate-risk level, where the moderate-risk zone dispersed in all parts of Burundi covering about 78.5% of the total land surface. The high-risk zone was found to be located mainly in the northern and western parts of Burundi covering about 9.1% of the total area of Burundi, which are characterized generally by a relatively low altitude, gentle slopes, high relative humidity and receive heavy rains. Low-risk zone, which represented about 12.4% of the total land surface, was found to be distributed in the eastern and southern parts of Burundi. The accuracy of the risk map was assessd and it showed that the high-risk zone captured 65% of the total cases of malaria that are reported at district level in Burundi in 2010. | Hassaan et al, 2017 |
|  |  |  |  |  |  | Expected changes in climate variables ( temperature and rainfall) under different scenarios will have significant impact on malaria incidence, especially in eastern parts that are expected to experience higher levels of warming and the northern parts that are expected to experience wetter conditions. The future malaria risk map shows that high-risk zone is expected to expand generally in the northern and eastern parts of Burundi, it is expected that high risk zone will expand to cover about 34.6% and 44% of Burundi land sur- face by 2050 under RCPs 2.6 and 8.5 scenario, respectively. While mod- erate and low-risk zones are expected to cover together 65.4% and 65% of Bu- rundi land surface by 2050 under RCP 2.6 and RCP 8.5 scenarios, respectively. It is expected that about 37% and 47% of Burundi are expected to experience exacerbated risk level under RCPs 2.6 and 8.5 scenarios by 2050. |  |
| Mali | Food security | Malnutrition | 14,238 children | The study conducted a multivariate linear regression analysis through the spatial coupling of FEWS NET climate data and DHS health data, using the Famine Early warning System Network (FEWS NET) trend analysis and the FEWS NET climatology (FCLIM) observation trends to predict future climatic changes, and using temperature and precipitation as environmental predictors. Three commonly utilized measures of nutrition were selected from the DHS for analysis: the child’s level of anaemia, the child’s measure of stunting, and the child’s measure of underweight status. | 2010 - 2050 | The FEWS NET trend analysis maps for JJAS rainfall and air temperatures shows that pockets of rainfall reduction appear near the border of Senegal and Mali. In visualizing climate change across Mali using the combined temperature and precipitation index transects (PPET), they showed the spatial transition from wet-cool southwest to hot-dry northeast. The 2010-2025 projected transects show a pattern of decreasing rainfall in the West with a contrasting increase in the East. For both temperature and rainfall, the interpolated changes were significant, suggesting that drying and warming have shifted semi-arid climate zones southward. In malnutrition modelling, results indicate that more arid regions were correlated with worse nutritional measures, and less densely populated areas (rural) did not show higher prevalence of malnourishment than high density areas (urban). But the study suggests that anaemia is diminished in arid climates due to livelihoods that largely rely on meat rather than grain consumption, but it negatively affects underweight and for stunting, arid climate trends influence malnutrition even when controlling for livelihood effects, suggesting a climatic effect on malnutrition beyond the adaptability and coping mechanisms of livelihoods. Finally, the study shows by 2025 approximately one quarter of a million children will suffer stunting and nearly two hundred thousand will be malnourished in the expanding arid zone. | Jankowska et al, 2012 |
|  |  |  |  |  |  | Results suggest that cluster measures of underweight and anaemia appear influenced by livelihoods and mitigation efforts should therefore focus on livelihood adaptation strategies. However, stunting, a chronic outcome of malnutrition, is influenced by climate in addition to livelihood |  |
| South Africa, Cape town | Non-communicable diseases and air-borne diseases | Respiratory diseases and cardiovascular diseases | 58,818 for CVD, and 54,317 for RD | A quasi-Poisson regression model was used to investigate the association between temperature variability (TV) and health outcomes (CVD and RD) based on hospital admissions | 2011 - 2016 | The study found positive and statistically significant associations between TV and hospitalisations for both diseases. The highest increase in CVD hospitalisations was observed at 0–3 days of exposure, while RD hospitalisations reached a peak after 2 days. When the model was controlled for the effects of daily mean temperature, the highest effect estimates appeared at 0–2 days for CVD hospitalisation, and at 0–1 day for RD hospitalisation. | Makunyane et al, 2023 |
| South Africa, Limpopo province | Extreme heat exposure | Heat-related illnesses (sunstroke, heat cramps, heat exhaustion, and heat stroke) | 406 households | The study obtained data for hourly measurements for daily ambient temperature (AT), humidity, and wind speed, and recorded temperatures and relative humidity for each season (spring, summer, winter) in the households, and hourly apparent temperatures were calculated. Multiple linear regression was used to identify the most important predictor of indoor AT by examining the association between indoor AT, ambient outdoor temperature and AT. Linear regression was used to predict indoor AT based on predicted outdoor temperature based on the projected increases in monthly temperature for SA under the RCP8.5 climate model for 2080-2099. Statistical tests were also used to examine the differences in temperatures according to roof type between indoor and outdoor AT. | 2088 - 2099 | Utilizing USNWS NOAA heat index, the study found that daily indoor AT was within each of the symptom bands (Caution, Extreme caution, Danger, Extreme danger), with 61 days in the more severe extreme caution bands, and the AT generally appeared to fall within the caution and extreme caution symptom bands from mid-morning to early afternoon during all seasons. AT was also classified as being in the danger symptom band between 11:00 a.m. and 6:00 p.m. in several homes during the summer season, and in winter the caution symptom band was the highest one to which AT rose. The multilinear regression results show that that outdoor temperature was most statistically significant in explaining indoor AT. The linear regression model predicted that indoor AT will increase significantly in spring and winter, compared to summer, putting people in higher risk of adverse health effects due to high AT during previously cooler seasons of spring and winter. Additionally, the study shows that there are more days during which predicted future indoor AT falls within the caution and extreme caution bands compared to the number of days during which observed AT falls within those same bands, it also predicts that there will be fewer days where household occupants will be exposed to to ATs that don't pose a health risk, indicating an overall increased risk to high AT. | Kapwata et al, 2018 |
| Global | vector-borne diseases, food security and water-borne diseases | Malaria, malnutrition (stunting and wasting), diarrhoeal diseases |  | The study calculated the cost of treatement for the 3 climate-sensitive diseases under 3 climatie scenarios in 2030 by multiplying the numbers of current cases by the relative risks for climate change, and then multiplied the outcome by the current costs of treatment per case. It employed data from the current number of cases for the three climate-sensitive diseases, the projected relative risks associated with climate change, and published data one the current costs of treatment for these diseases, under the assumption the cost would remain constant, and it didn't account for socioeconomic development. | 2030 | The study found that the largest climate change-attributable cases are projected to be in Africa and Southeast Asia. The projected excess costs for managing excess cases of the 3 health outcomes under emissions reduction scenario resulting in stabilization at 550 ppm CO2 (s550) are $3,333 to $10,689 million US$. Under the emissions reduction resulting in stabilization at 750ppm CO2 (s750) are $3,992 to $12,603 million, and under the unmitigated emission scenario are $5,852 to $17,957 million. The study concludes that under s750 scenario, the annual needs would for managing these diseases will be almost as much as the current total annual overseas development assistance for health. It estimated that in 2030, the total investment needs to manage diarrhoeal diseases will be $67 billion, malnutrition $2 billion, and $36 to $50 billion for malaria. | Ebi L., 2008 |
| Ghana | food security |  | 84 participants | Qualitative stuady, semi-structured interviews and focused group discussions that focused on the effects of climate change on households' livelihood, adpatation practices and the effects of remittance on financing adaptation | 2016 | The study provided evidence that worsening sea level rise and coastal riverine erosion have confsicated participants homes and livelihood sources. It identified local adaptation measures such as delaying sowing to offset adverse effects of potential climate variability, but struggled with access to climate information to inform optimal adaptation strategies. Several local adaptation measuer were revealed, such as using indigenous knowledge systems, multi-cropping as a traditional adaptation strategy, modern adaptation techniques (agroforestry management, seed production, energy saving, bushfire management) that incorporate the use of climatic information. The study noted that the cost of adaptation among the respondents was significantly high, such as the cost of relocation, the cost of rebuilding perceived climate change-induced property destruction, The majority of the respondents demonstrated the lack of required resource-sets to adapt to climate change effectively. In examining how the participants financed these climate-induced costs using remittances, the study found that they focused on specific risk diversification activities, purchasing basic needs and noting its substantial impact on households’ nutrition and health, acquiring equipment to aid in building adaptive capacity, and technological tools such as mobile phones to support their farming activities. Noting that remittances were a major source of financing adaptation to climate change. | Musah-Surugu et al, 2017 |
| Africa | Health risks due to extreme weather events (temperature) | Heat stroke, heat cramps, or heat exhaustion |  | The study used a CCAM model to project changes in extreme apparent temperature over the African region by running the model with a 200 km quasi-uniform resolution over the whole globe using sea-surface forcing from different global climatic model, and then adjusted the model to capture the African region. The apparent temperature was calculated using the bias-corrected relative humidity and maximum temperature fields. The projected AT was categorized into hot days, and based on symptom bands to illustrate the potential for direct healt-related health impacts. | 1961 - 2100 | The study projected that there will be an increase in the number of hot days, where the temperature exceeds the ATmax = 27 °C, which is used as a threshold to classify hot days across the continent. The potential risk of health to be impacted by high temperatures is projected to increase as well. The study reported that the high-lying areas of the escarpment from Ethiopia through Tanzania is projected to experience the highest increases in 2071-2100, and the highlands in East Africa are projected to see the largest average rate of increase in days per year over the full timescale. While there is less spatial variability in the increases projected for northern Africa, and more heterogeneity in the projected increases for southern Africa. Additionally, the majority of Africa is projected to have, on average, over 5 months of the year where the temperature is above the ATmax. The study didn't find any projected decrease in number of days for any of the 3 thresholds of hot days, and it identified that for equatorial Africa, and the western part in particular there will be numerous days above the Atmax = 32 °C threshold, this seen in the band from Angola, across northern Zambia, southern Democratic Republic of Congo and Tanzania, up across Uganda, Kenya, and Ethiopia, which will result in shifting a large number of days to the upper threshold. Only few countries are projected to experience an increase in hot days at the highest threshold of ATmax > 51 °C by 2100, such as Democratic Republic of Congo and eastern Sahara desert. Finally, the study concluded that the largest projected increases are occurring in the AT range of 27 °C ≤ ATmax ≤ 32 °C, and at all areas when an increase is projected, the increase was determined to be statistically significant. | Garland et al 2015 |
|  |  |  |  |  |  | In the symptom bands category, the study found that the decrease in days in the sympotm bands is due to the days moving up to more severe symptom bands. It identified that southern Africa are projected to see increases in days in Symptom Band II, and the Western Sahara is expected to see increases in days in the more severe symptom band. |  |
| Mozambique | vector-borne diseases, food security | Malaria, chronic and acute malnutrition | 162 district | This study employed the WHO's vulnerability and adaptation assessment (VAA), to examine vulberability, it used the vulnerability assessment model using exposure, sensitivity, and adapative capacity dimensions to compute HVI. It combined data on cliamtic variables (temperature, rainfall and relative humidity), epidemiologic data on annual cases of malaria, tuberculosis, HIV, chronic and acute malnutrition. It also integrated socio-economic data such as the literacy rate, the number of health workers, and data on food security. Following thtat, the HVI and its sub-indices (exposure index, sensitivity index, and adaptive capacity index) were classified into quintiles, and five categories of classification were created. | 1970 - 2016 | The study calculated exposure scores by examining climatic variables and extreme events. It concluded that heating is more evident in the southern part of the country, while the coastal provinces experience both extremes of heating and reduction of temperature, and inlands districts experience reduction in temperature. It found that majority of ditricts experienced an increase in the amount of rainfall. In studying patterns of extreme events (cyclones, droughts, and floods), the study determined that tropical cyclones affected coastal districts. It also found that 72 districts in the country were affected by droughts, particualrly the southern region. And for floods, the study found that all districts have been affected at some point by floods, particualry those around the river basins. | Muleia et al, 2023 |
|  |  |  |  |  |  | On the exposure index, the study identified the southern region to be in a conserably higher exposure to climatic hazards. |  |
|  |  |  |  |  |  | On the sensitivity index, it was found that sensitivity to cimatic hazards was low in the northern region, compared to being modrate in the centre and south regions. Lastly, on the adaptive capacity index, it was found that the southern region had the highest capaicty compared to other regions. |  |
| South Africa | Vector-borne diseases, water-borne diseases | Malaria, Diarrheal diseases | 20 | Key expert interviews with African experts in the fields and intersections of climate change and health. A thematic content analysis was conducted. | 2019 | The interview findings were classified into thematic categories. In the vulnerability and risk category, the participants touched on individual attitudes and knowledge about climate change and the media's role, they emphasized on the need to prepare for the mental health and physical vulnerability. The experts also weighed on the geographic, household, health and community vulnerability by pointing out the heightened vulnerability of specific regions such as KwaZulu-Natal and Eastern and Western Cape, they also noted on the link between climate change and the change in malaria vector patterns, heightened diarrhoeal disease and plague, along with its potential negative impact on components of the health system, such as infrastructure, supply chain issues, and medical services. Taking in consdieration the lack of adequate services. They brought further attention to the lack of resources available to effectively cope with climatic crises, and the vulnerability on the household level among the poorest and other vulnerable groups. | Dos Santos et al, 2022 |
|  |  |  |  |  |  | In the adaptation category, the experts recommended designing climate change-resilient health systems, with measures such as cooling systems, and facilitating transport. They touched on information availability and synergy between stakeholder at different levels, along with ethical accountability. Emphasis was placed on linking research and evidence-based policy decisions. The study listed a number of assessmnet tools and frameworks that are currently being used to adapt to climate change. Additionally, the study identified the different roles of stakeholders in developing health risk assessments. It also highlighted the need to design the NHI with the impacts of climate change on health in mind. Finally, the study illustrated the importance of local ownership, along with faced barriers such as lack of proritization of the health sector and the impact of climate hazards on health. |  |
| South Africa | Food security, waterborne diseases, and extreme weather events | Malnutrition, | 234 Health professionals (doctors, medical students, and allied health professionals) | The study conducted a Fisher's exact test to assess the association between the health professionals demographics and their perspectives on climate change and its impact on healthcare. Data was collected through distributing a national suvery among various healthcare professionals, it focused on views on climate change, perceptions on climate change and its impact on diseases as well as preparedness of healthcare systems for climate change. | 2022 | The study showed that most of the participants viewed climate change as a reality, and almost two-third indicated that waterborne diseases and malnutrition are severly affected by climate change, while others touched on respiratory illnesses and mental health. Most participants were not aware if or how their facilities were adapting to climate change, but it was found that public healthcare workers are less likely to be aware, compared to private ones. | Manga et al, 2022 |
| Lagos, Nigeria, and South Africa | Water-borne diseases/ diarrheal diseases. Vector-borne diseases | Typhoid and Malaria | Ajegunle-Ikorodu Community | Analysis of 3 case studies in Lagos concerning community-based approaches to integrated governance of climate change and health. The study proposes a community-oriented model for integrated climate change and health action, based on a previous model in South Africa. | 2021 | The Ajegunle-Ikorodu Community Resilience Action Plan: aimed to develop early warning systems, flood mitigation measures, and community participation in flood adaptation. Despite high flood vulnerability, the community did not consider flooding a priority due to unfulfilled promises of institutional interventions. The community adopted several coping mechanisms, including collective action and the use of indigenous knowledge systems. The case study highlights the interdependency between flooding, health hazards, and adaptive practices. The study advocates for an integrated approach to climate and health resilience, considering the impact of flooding on health and seeking to adapt and mitigate against these health impacts. The case study underscores the significance of community-led interventions, social capital, and the agency of collective action in addressing climate and health resilience. The study also points out the need to integrate ground-level resilience and effective capacities into health services and governance. | Oni et al, 2021 |
| Nairobi, Kenya | Vector-borne diseases, waterborne diseases, food security, | NCDs, malaria, cholera | 28 participants working with climate change related health issues in the Mukuru informal settlement | A qualitative study in which 3 focused groups, and 5 in-depth interviews were conducted | 2021 | Climate change related diseases: the study discussed the impact of climate change on non-communicable diseases (NCDs) and other related challenges. It highlighted the connection between climate change and the prevalence of NCDs, such as cardiovascular diseases, respiratory issues, and mental disorders. It also emphasized the influence of climate change on food insecurity, rising food prices, malnutrition, and access to clean water. | Andersen et al, 2021 |
|  |  |  |  |  |  | Environmental risk factors: the study emphasized the significant impact of air pollution on non-communicable diseases (NCDs) in the context of climate change. It highlighted the prevalence of air pollution, both indoor and outdoor, in informal settlements, and its association with the development of NCDs such as asthma, cardiovascular diseases, and respiratory illnesses. Additionally, it underscored the adverse effects of indoor air pollution, contamination of food and water, and the lack of proper waste management on the health of individuals in these settings. |  |
|  |  |  |  |  |  | Urban planning and publich infrastructure: The study touched on the impact of infrastructure, urban planning, and environmental factors on the health of individuals in the context of climate change. It emphasized the lack of proper infrastructure, such as roads, drainage systems, and housing, in informal settlements, which contributes to various health challenges, including the vulnerability to climate change-related hazards. Additionally, the study highlighted the need for improved waste management, sewage systems, and the replanting of trees to address the adverse effects of climate change on public health. |  |
|  |  |  |  |  |  | Economic risk factors and vulnerable groups: The study emphasized the impact of economic risk factors and the vulnerability of specific groups in the context of climate change and health. It highlighted the association between poverty and the increased susceptibility to climate change-related health hazards, particularly in informal settlements, where access to quality healthcare is limited. It also highlighted the vulnerability of children, the elderly, and people living with non-communicable diseases (NCDs) to the adverse effects of climate change, such as waterborne diseases, respiratory issues, and other health challenges. |  |
|  |  |  |  |  |  | Adaptation strategies: The participants discussed potential adaptation strategies in the context of climate change and health. They emphasized the importance of urban planning measures, as well as the need for reforestation and planting of trees and vegetation. Furthermore, they touched on the significance of educational measures to empower residents in informal settlements and raise awareness about climate change and its impacts on health. Finally, they highlighted the need for government advocacy, coordination of efforts, and investment in climate change resilience in urban areas. |  |
| Niger | Airborne diseases (droplets) | Meningitis | the residents of Niger's 38 health districts | The researchers used wavelet and phase analysis methods to define and compare the time-varying periodicities of meningitis, climate, and dust in Niger. They focused on detecting time-lags between the signals that were consistent across districts. The epidemiological data consisted of reported number of suspected meningitis cases per week from 1986 to 2007, for Niger’s 38 health districts. The aerosol index (AI), a semi-quantitative index of the aerosol loads integrated over the whole atmospheric column, was used to measure dust levels. Climate variables considered were temperature, wind force, wind direction, and relative humidity. | 1986 - 2007 | The study's results highlighted the special case of dust in comparison to wind, humidity, or temperature. A strong similarity between districts was noticed in the evolution of the time-lags between the seasonal component of dust and meningitis. This result, together with the assumption of dust damaging the pharyngeal mucosa and easing bacterial invasion, reinforces the confidence in dust forcing on meningitis seasonality | Agier et al, 2012 |
| Nigeria, Zimbabwe, Madagascar, Burkina Faso, Bangladesh, and Sierra Leone | Mental health |  |  | Collected reports and prepared case studies from country offices of a NGO that supports mental health and psychosocial support (MHPSS) programmes in these countries. The case studies documented the context, the impacts of climate-related events or gradual change, the programme/intervention delivered in response, and the key lessons that we were able to learn from the experience. | 2019 | Nigeria: The project aimed to rebuild destroyed health infrastructure to facilitate the return of communities. The activities were rebuilding and re-equipping of destroyed health centres, training, retraining and support of healthcare workers to be able to integrate mental healthcare into the services in the health centres, training and support of family members, Village Health Workers, Junior Community Health Extension Workers and community volunteers to support persons with mental health problems in the community, provision of essential medicines and establishment of Drug Revolving Funds, provision of water, sanitation and hygiene in the community, support of livelihood opportunities, ensuring inclusion of persons with disability in the community, for example, being members of the Water Committee and training to maintain the water pumps. And finally, raising awareness on the availability of mental health services. | Eaton et al, 2022 |
|  |  |  |  |  |  | Zimbabwe: The project aimed to build the capacity of nurses and key community stakeholders on MHPSS to support the wellbeing of individuals and communities affected by Cyclone Idai and COVID-19. The activities were focusing on improving service delivery of mental healthcare and improving access to services through community awareness and training in MHPSS, targeted training for primary healthcare workers based on the mhGAP-Humanitarian Intervention Guide, build the capacity of local community influencers to improve MHPSS, and engaged communities to strengthen existing support support structures. |  |
|  |  |  |  |  |  | Bukrina Faso: The project addressed food and nutritional security of vulnerable households with the aim of sensitizing all stakeholders to sustainable management of natural resources in the context of the effects of climate change. The activities focused on Improving knowledge and practices of municipalities in terms of climate risks and integrated management of natural resources, strengthening MHPSS, including self-help groups, training of health and community workers on the management of conditions linked to stress aimed at ensuring comprehensive care in nonspecialised health centres using mhGAP, and training of health workers and traditional healers on the rights of people living with a psychosocial and intellectual disability |  |
| India |  |  | Indian villages in 8 states (Ghazipur, Uttar Pradesh, Haryana, Rajasthan, Telangana, Andhra Pradesh, Karnataka, Tamil Nadu) | Save the Children International in India partnered with Nokia and Digital Empowerment Foundation to role out smart villages across India by integrating technology in existing practices across health, education, livelihood, governance, finance, and entertainment sectors. | 2018 - 2023 | The health pillar of the Smartpur initiative aims to provide accessible and affordable medical diagnosis and consultation through telemedicine. Efforts under this pillar include setting up a telemedicine and diagnostic kit center, collaborating with local hospitals and doctors, and designing awareness campaigns on the importance of early diagnosis and preventive measures related to water, sanitation, and hygiene. The initiative also involves training community stakeholders in digitally accessing health-related information for better health governance. Smartpur has collaborated with an online platform of over 300 doctors called DocOnline to provide medical health facilities and medical tests to Smartpur villages via telemedicine. The initiative also seeks to address the problem of inadequate access to quality healthcare and poor awareness of preventive healthcare measures at the village level by encouraging good health and hygiene practices and maintaining medical histories. Rural entrepreneurs will work with community members, frontline health workers, and staff of primary health centers and community health centers to implement these efforts. The initiative also aims to address the overdependence on unpaid or poorly paid community health workers, particularly women, by adapting existing community health worker structures for integrated climate and health action. The efforts under the health pillar emphasize the importance of leveraging telemedicine and digital tools to improve access to healthcare and promote preventive measures in rural areas. | <https://smartpur.in/the-project/> |
| Coastal Kenya | Vector-borne diseases | Malaria | Residents in rural region in Kilifi county, Kenya | The study considered two climatic variables, rise in air temperature and elevated atmorpheric CO2 concentration. The researchers used a stochastic lattice-based malaria (SLIM) model to predict changes in Anopheles vector abundance, the life cycle of Plasmodium parasites, and thus malaria transmission under projected climate change in the study region. The SLIM model incorporated a non-linear temperature-dependence of malaria parasite development to estimate the extrinsic incubation period of Plasmodium. It was also linked with a spatially distributed eco-hydrologic modelling framework to capture the impacts of climate change on soil moisture dynamics, which served as a key determinant for the formation and persistence of mosquito larval habitats on the land surface. | 2008 - 2013 | The study found that under elevated atmospheric CO2 concentration only, modeled results revealed wetter soil moisture in the root zone due to the suppression of transpiration from vegetation acclimation, which increases the abundance of Anopheles vectors and the risk of malaria. When air temperature increases were also considered along with elevated CO2, the life cycle of the Anopheles vector and the extrinsic incubation period of Plasmodium parasites were shortened nonlinearly. However, the reduction of soil moisture resulting from higher evapotranspiration due to air temperature increase also reduced the larval habitats of the vector. The study concluded that vegetation acclimation triggered by elevated CO2 under climate change increases the risk of malaria, and that the indirect impacts of temperature change on soil moisture dynamics are significant and should be weighed together with the direct effects of temperature change on the life cycles of mosquitoes and parasites for future malaria prediction and control | Le et al, 2019 |
